# The Structure of *Spiroplasma Virus 4*: Exploring the Capsid Diversity of the *Microviridae*

**DOI:** 10.3390/v16071103

**Published:** 2024-07-09

**Authors:** Mario Mietzsch, Shweta Kailasan, Antonette Bennett, Paul Chipman, Bentley Fane, Juha T. Huiskonen, Ian N. Clarke, Robert McKenna

**Affiliations:** 1 Department of Biochemistry and Molecular Biology, College of Medicine, Center for Structural Biology, McKnight Brain Institute, University of Florida, Gainesville, FL 32610, USAdendena@ufl.edu (A.B.);; 2 The BIO5 Institute, Keating Building, University of Arizona, Tucson, AZ 85721, USA; 3 Institute of Biotechnology, Helsinki Institute of Life Science HiLIFE, University of Helsinki, 00014 Helsinki, Finland; 4 Molecular Microbiology Group, Faculty of Medicine, University of Southampton, Southampton General Hospital, Southampton SO16 6YD, UK

**Keywords:** *Microviridae*, bacteriophage, cryo-EM, capsid, *Spiroplasma virus 4* (SpV4), chlamydia phage 2 (ChP2)

## Abstract

*Spiroplasma virus 4* (SpV4) is a bacteriophage of the *Microviridae*, which packages circular ssDNA within non-enveloped T = 1 icosahedral capsids. It infects spiroplasmas, which are known pathogens of honeybees. Here, the structure of the SpV4 virion is determined using cryo-electron microscopy to a resolution of 2.5 Å. A striking feature of the SpV4 capsid is the mushroom-like protrusions at the 3-fold axes, which is common among all members of the subfamily *Gokushovirinae.* While the function of the protrusion is currently unknown, this feature varies widely in this subfamily and is therefore possibly an adaptation for host recognition. Furthermore, on the interior of the SpV4 capsid, the location of DNA-binding protein VP8 was identified and shown to have low structural conservation to the capsids of other viruses in the family. The structural characterization of SpV4 will aid future studies analyzing the virus–host interaction, to understand disease mechanisms at a molecular level. Furthermore, the structural comparisons in this study, including a low-resolution structure of the chlamydia phage 2, provide an overview of the structural repertoire of the viruses in this family that infect various bacterial hosts, which in turn infect a wide range of animals and plants.

## 1. Introduction

*Spiroplasma virus 4* (SpV4) is a bacteriophage of the genus *Spiromicrovirus*, in the subfamily *Gokushovirinae*, within the *Microviridae* [1]. It is a bacteriophage of *Spiroplasma melliferum* and was first found in *S. melliferum* cultured from honey bees [2]. Spiroplasmas are a group of small, cell-wall free bacteria belonging to the class Mollicutes. These bacteria can act as pathogens or endosymbionts of arthropods, vertebrates, and plants. *S. melliferum* are pathogens of honeybees and are likely the causative agents of a neurological disease in bees termed “spiroplasmosis” and “May disease” [3,4].

The virion of SpV4 is composed of a non-enveloped T = 1 icosahedral capsid that packages a ~4.4 kb circular, single-stranded DNA (ssDNA) genome. Nine open reading frames (ORFs) are present in the SpV4 genome [5]. ORF1 expresses viral protein 1 (VP1), with a molecular weight of ~62 kDa, which is the major capsid protein. Previously, the capsid structure of SpV4 has been determined at 27 Å resolution by cryo-electron microscopy (cryo-EM) and shown to have a capsid diameter of ~270 Å assembled from 60 copies of VP1 [6]. At that resolution, mushroom-like protrusions were observed, projecting an additional ~50 Å outward from the 3-fold symmetry axes. The capsid protein of SpV4 is predicted to possess an eight-stranded antiparallel beta-barrel core structure, similar to the capsids of phiX174 [7], phiEC6098 [8] within the *Microviridae*, and to other ssDNA virus families [9,10]. The interconnecting loops located between the β-strands form the surface topology of the capsid and display the lowest amino acid (aa) sequence variability between the VP1 of the *Gokushovirinae* [1,11]. Within the subfamily *Gokushovirinae*, SpV4’s capsid protein shares an aa sequence identity of the capsid protein of ~30–36% compared to the chlamydia phages (ChP) 1–4, Escherichia phage phiEC6098, and Bdellovibrio phage phiMH2K that are assigned to the genera *Chlamydiamicrovirus*, *Enterogokushovirus*, and *Bdellomicrovirus*, respectively. By contrast, the capsid sequence identity to the well-characterized phiX174 phage, which is a member of the subfamily *Bullavirinae* in the *Microviridae*, is only ~15% [7].

**Figure 1:**
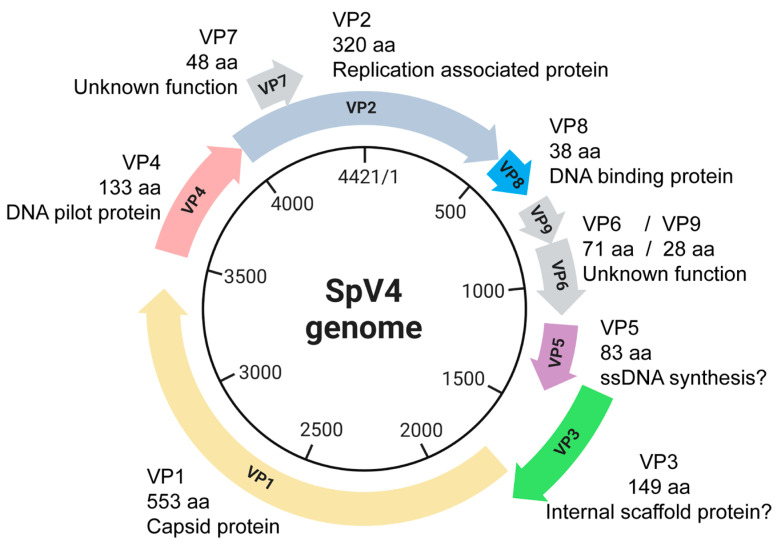
The circular, ssDNA SpV4 genome (4421 nt) and its protein products.

Some of the remaining ORFs 2–9 encode proteins with functions analogous to those in phiX174 (Figure 1). For example, VP2 encodes the protein A homologue seen in the phiX174-like viruses which orchestrates genome replication [12,13]. Despite low sequence homology to known scaffolding proteins, VP3 behaves like the phiX174 internal scaffolding B-protein [14]. VP4 is a glycine-rich protein with a predicted helical fold that is strongly consistent with the known structure of the phiX174 H-protein, which forms a DNA translocating tube during penetration [15,16]. VP5 encodes for a small protein, which may be analogous to the phiX174 C-protein, which regulates the switch from dsDNA replication to ssDNA genome biosynthesis [17]. VP6, 7, and 9 are non-structural proteins that are not functionally characterized due to their lack of sequence similarity to known proteins. Lastly, VP8 encodes for an arginine–lysine-rich polypeptide that is analogous to the J-protein of phiX174 and is important for DNA packaging [18]. The VP8/J-protein has been observed in the capsid structures of the bacteriophages phiX174, α3, G4, and phiEC6098 [7,8,19,20].

In this study, the capsid structures of genome-containing and empty SpV4 virions were determined to 2.5 and 3.0 Å resolution, respectively, using cryo-electron microscopy (cryo-EM) and three-dimensional (3D) image reconstruction. The mushroom-like protrusions of the SpV4 capsids became increasingly disordered at higher resolutions with imposed icosahedral symmetry. Hence, localized reconstruction of these protrusions was employed and resolved this region to ~4 Å resolution. The VP8, DNA-binding protein was observed in the interior of both genome-filled and empty SpV4 capsids. In addition, the capsids of SpV4 were compared to low-resolution reconstructions of the ChP2 capsids and the structural repertoire of the *Microviridae* was analyzed.

## 2. Materials and Methods

### 2.1. Propagation and Purification of SpV4 and ChP2

SpV4 particles were propagated and purified as previously described [6]. Briefly, SpV4 was propagated in the *S. melliferum* strain G1 grown at 32 °C. The SpV4 virions were purified by pelleting the virus through a 30% sucrose cushion by centrifugation (130,000× *g* for 7 h in a Beckman SW27 rotor, Beckman, Brea, CA, USA). The pellet was resuspended in 50 mM sodium tetraborate buffer containing 0.6 g/mL CsCl and centrifuged to equilibrium at 150,000× *g* for 48 h. The fraction containing the SpV4 virions was collected and dialyzed against 40 mM sodium tetraborate buffer at pH 9.2. The virus concentration was adjusted to 1 mg/mL.

ChP2 particles from Chlamydophila abortus (strain MA) were propagated and partially purified as previously described [14,16]. These particles were further treated with an equal volume of chloroform and incubated for 30 min at 4 °C followed by centrifugation at 9200× *g* for 10 min at 4 °C to remove membranous material. The particles in the aqueous phase were collected and used for these studies.

### 2.2. Vitrification and Cryo-Electron Microscopy Data Collection

For vitrification, the Vitrobot Mark IV (FEI) automatic plunge-freezing system was utilized. Three microliters of SpV4 or ChP2 were applied to glow discharged C-flat holey carbon-coated grids (Protochips Inc, Morrisville, NC, USA). The sample was incubated on the grids at 4 °C and 95% humidity for 3 s prior to blotting using filter paper and plunging into the ethane slush. Subsequently, the grids were maintained at liquid nitrogen temperatures until data collection. The grids were screened in-house or, in the case of ChP2, imaged using an FEI Tecnai G2 F20-TWIN microscope (FEI Co., Hillsboro, OR, USA) operated under low-dose conditions (200 kV, ~20 e^−^/Å^2^). The high-resolution cryo-EM data collection for the SpV4 samples was performed at the University of California, Los Angeles (UCLA) using a Titan Krios electron microscope (Thermo Fisher, Waltham, MA, USA). The microscope was operated at 300 kV and data were collected on a K3 (Gatan, Pleasanton, CA, USA) direct electron detector camera. The Titan Krios electron microscope was equipped with a Gatan Energy Filter and a slit width of 20 eV was set for the energy filter. Thirty image frames were recorded in counting mode, with the dose rate of 34 e^−^ per Å^2^ on the sample. MotionCor2 (Version 1.5.0) was used for aligning the movie frames collected on the K3 detector with dose weighting [21]. The data were collected as part of the National Institutes of Health (NIH) “West/Midwest Consortium (WMC) for High-Resolution Cryo Electron Microscopy” project.

### 2.3. Data Processing and 3D Image Reconstruction

The cisTEM software package (Version 1.0.0) was utilized for the 3D image reconstruction of the data sets as previously described [22]. Briefly, the aligned micrographs were imported into the program and their CTF parameters estimated. The CTF information was used to eliminate micrographs of poor quality. This was followed by automatic particle picking using a particle radius of 135 Å. This set of particles was subjected to 2D classification that eliminated non-capsid particles from the automatic picking process and separated empty and genome-filled capsids. Subsequently, the capsids of SpV4 or ChP2 were reconstructed using default settings using icosahedral averaging. This included the ab initio 3D model generation, auto refinement, and density map sharpening with a pre-cutoff B-factor value of −90 Å^2^ and variable post-cutoff B-factor values of 0, 20, and 50 Å^2^. The sharpened density maps were inspected in Chimera (https://www.cgl.ucsf.edu/chimera/) [23]. The resolutions of the cryo-EM maps were estimated based on a Fourier Shell Correlation (FSC) of 0.143. For the determination of the local resolution, the ResMap application (version 1.95) was utilized [24].

### 2.4. Localized Reconstruction

The stack of individual particle images and the parameter file were exported in cisTEM to the Relion format. All further steps were conducted in Scipion3 [25]. The 77,204 individual particle images from the icosahedral reconstruction were imported and the subparticles defined by specifying a vector length of 140 Å from the center of the particle along the 3-fold symmetry axis (protocol: localrec—define subparticles), as described before [26]. In the subsequent step, the subparticles (~4.6 million) were filtered, keeping only unique subparticle images and removing view angles that significantly overlap with capsid (protocol: localrec—filter subparticles, more than 35° deviation from the side view). The individual images of the remaining 773 K subparticles were extracted (protocol: localrec—extract subparticles) and used for the reconstruction of an initial low-resolution map with a C1 symmetry operator (protocol: Relion—reconstruct). This map was then used as an input volume for the 3D classification protocol using four classes and Blush regulization. During this step, resolution in the expectation step was limited to 12 Å and relaxation of the original C3 symmetry was applied. No additional alignment was performed. The resulting maps of the individual classes were inspected. The map and particles of the class showing ordering of the 3-fold protrusion were selected for further refinement using the Relion 3D auto-refine protocol using local alignment (sigma of the orientational prior of 5 degrees) with Blush regularization. The final map (based on 249 K subparticles) was sharpened using the Relion post-processing protocol.

### 2.5. Model Building

AlphaFold3 was used to generate in silico models of SpV4, ChP2, and phiMH2K VP1 based on the primary aa sequence [27]. Using ViperDB (https://viperdb.org/), full icosahedrons (60-mer) using these models were generated [28]. These were docked into the cryo-EM density map with Chimera using the “Fit in Map” option [23] and the voxel size was adjusted to maximize the correlation coefficient. The EMAN2 subroutine e2proc3d.py was utilized to resize the maps based on optimized voxel size as determined by correlation coefficients from Chimera and converted to the CCP4 format using MAPMAN (version 7.8.5) [29]. The main and side chains of the SpV4 model were manually refined in Coot using the real-space refinement tool [30]. The SpV4 model was further automatically refined using PHENIX (version: 1.10.2155), which also provided the final refinement statistics (Table 1) [31].

### 2.6. Structural Comparison

For structural comparison, the SpV4 VP1 model was superposed in Coot (version: 0.8.9.1) onto the previously determined structures of phiX174 (PDB ID: 2BPA), α3 (PDB ID: 1M06), G4 (PDB ID: 1GFF), and phiEC6098 (PDB ID: 8DES) to obtain the distances of the aligned Cα positions. Distances between non-overlapping Cα positions, due to residue deletion/insertions, were measured using the distance tool in Coot. Regions of two or more adjacent aa with ≥2.0 Å difference in superposed Cα positions were considered to be structurally diverse. Subsequently, the structural similarity was calculated as the number of aa within 2 Å divided by the total number of aa. For the sequence identity, the primary aa sequences were compared using Blastp (https://blast.ncbi.nlm.nih.gov/).

## 3. Results and Discussion

### 3.1. Purified SpV4 Contains Two Particle Populations: Empty and Full Capsids

**Figure 2:**
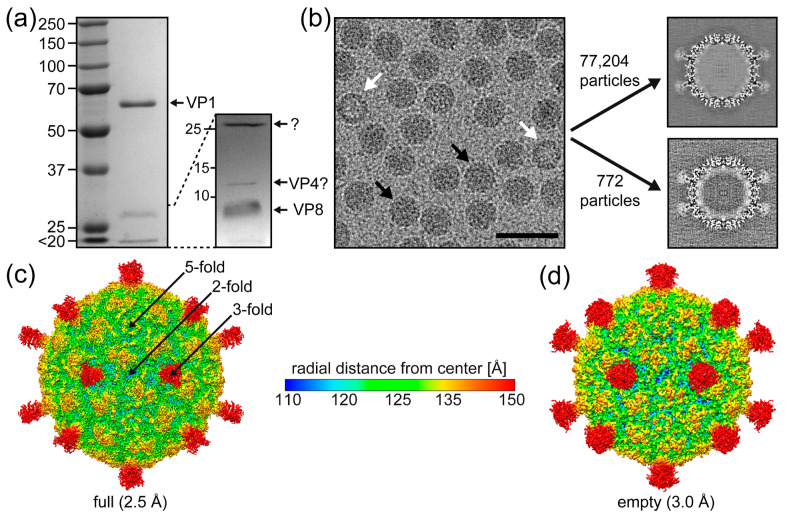
The SpV4 capsid structure. (**a**) A 9% SDS-PAGE of purified SpV4, with a band at ~62 kDa equivalent to the size of VP1. A 15% SDS-PAGE resolves the low molecular weight bands. (**b**) Cryo-electron micrograph of the SpV4 sample. Black arrows indicate genome-filled capsids, whereas white arrows indicate empty capsids. Scale bar: 500 Å. Orthogonal slices of the final maps for “full” and “empty” capsids are displayed. (**c**) The capsid surface density maps of genome-filled (“full”) and (**d**) empty SpV4 capsids contoured at a sigma (σ) threshold level of 1.0 are shown. The maps are radially colored (blue to red) according to distance to the capsid center, as indicated by the scale bar. The approximate icosahedral 2-, 3-, and 5-fold axes are indicated.

The purified SpV4 sample analyzed by SDS-PAGE gel showed a major band at ~60 kDa, consistent with the size of SpV4 VP1 of 62 kDa (Figure 2a). Additional weaker bands of lower molecular weight were observed. Between 10 and 15 kDa, a band was observed where VP4 is expected to migrate. In phiX174, the equivalent H-protein is incorporated at 10–12 copies per virion and could explain the ratio of 1:5 to 1:6 relative to VP1 [32]. At ~5–10 kDa, a band was detected, which is likely the highly positive-charged VP8 (5 kDa) proteins of SpV4. Due to its charge, VP8 is expected to migrate higher than its molecular weight. Between ~25 and 30 kDa, another band was observed, which could be host cell contaminant proteins or cleavage products of VP1.

Cryo-EM micrographs showed intact particles of ~270 Å in diameter with either a dark (genome-containing = “full”) or light (packaging no ssDNA “empty”) interior appearance (Figure 2b). Thus, the SpV4 sample was deemed suitable for high-resolution structure determination by cryo-EM. The empty and full capsids were separated by 2D classification and subsequently reconstructed independently. The full capsids outnumbered the empty capsids by a ratio of approximately 100:1. As a result, a total of 77,204 full and 772 empty capsids were extracted. Three-dimensional image reconstruction of these particles resulted in capsid density maps, with the previously observed mushroom-like protrusions [6] at the 3-fold symmetry axis at a resolution of 2.5 and 3.0 Å, respectively (Figure 2c). These protrusions appeared to have a larger volume for the empty capsids, but this is most likely due to the lower resolution and/or lower number of particles (Figure 2d). The 5-fold symmetry axis showed a pore with a diameter of 4.5 Å at its narrowest point. Depressed regions were found at the 2-fold symmetry axis, surrounding the 3-fold protrusions and the 5-fold pore. These depressions flank a raised region located between the 2, 3, and 5-fold symmetry axes. No major differences were observed between the full and empty SpV4 capsids, except for disordered density filling the interior of the full capsid, which is absent in the empty capsid map (Figure 2b).

### 3.2. Packaged Genome Orders the N-Terminus of Full Capsids

**Figure 3:**
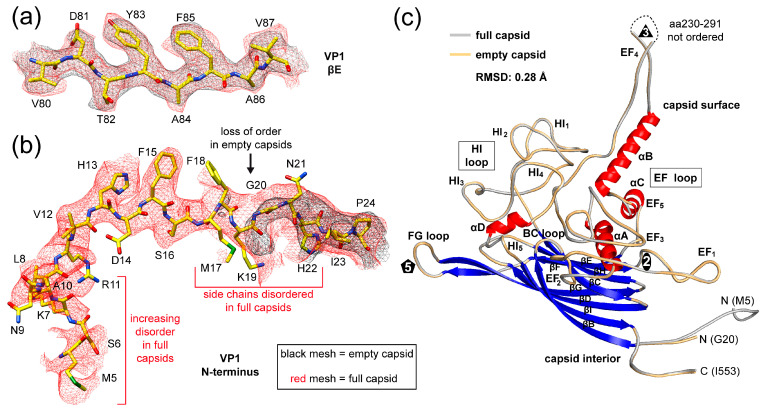
The SpV4 VP1 model. (**a**) Amino acid residues fit to the full capsids (in red) and empty capsids (in black) for the βE strand density maps are shown. The amino acid residues are shown in stick representation and colored according to atom type: C = yellow, O = red, N = blue, S = green. (**b**) Depiction as in (**a**) for the VP1 N-terminus. (**c**) Ribbon diagram of SpV4 VP1. The β-strands (blue, βB–βI), the α-helices (αA–D) helix, the N- and C-termini, and the approximate icosahedral 2-, 3-, and 5-fold axes are indicated. The main surface loops, including their subloops, are labeled.

The cryo-EM maps for the full and empty SpV4 capsids showed generally well-ordered densities for the amino acid side chains, allowing reliable model building and fitting (Figure 3a and Figure S1). The observed order in the density map for the full capsids started at methionine 5 (Figure 3b) to the C-terminal isoleucine 553. An exception to the structural ordering is the surface loop at the 3-fold symmetry axis comprising aa230–291, for which only diffuse density was observed, preventing the placement of the polypeptide sequence (Figure 3c). In the empty map of SpV4, structural order started at glycine 20 but the overall VP1 topology was identical, within the experimental parameters, to the full capsids with an Cα-RMSD of 0.28 Å. The reduced ordering of the N-terminus in the empty capsid is likely the result of the absence of the packaged viral genome. The N-terminus of SpV4 (aa1–19) is highly positive charged with a calculated isoelectric point of 10.6 due to the presence of five lysines, one arginine, and one histidine. Thus, the negatively charged ssDNA might stabilize the N-terminus in full capsids. Similar effects have been previously observed in other ssDNA viruses [33].

### 3.3. The SpV4 Capsid Surface Is Dominated by Two Loops

**Figure 4:**
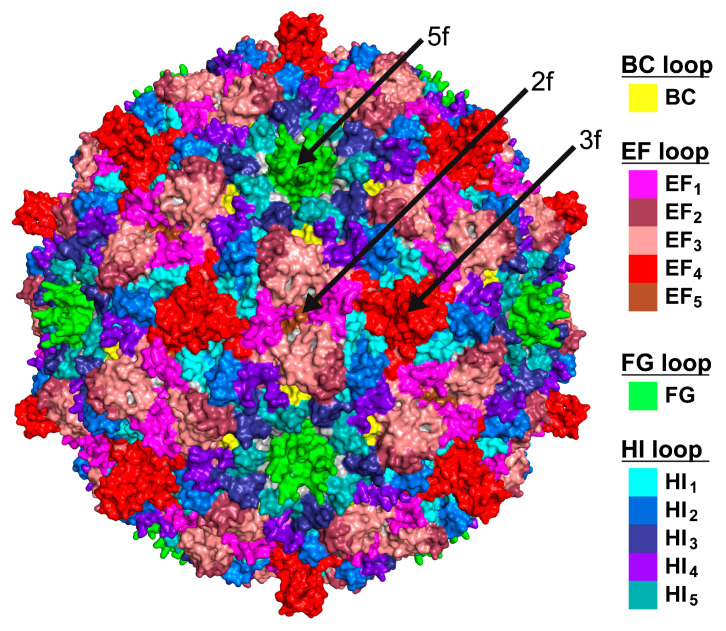
The SpV4 surface loops. Three-dimensional capsid surface representations, with the locations of the SpV4 surface loops colored. The positions of the icosahedral symmetry axes are indicated.

The SpV4 VP1 structure consists of a core, eight-stranded, anti-parallel β-barrel motif, with β-BIDG forming the inner surface of the capsid and the β-CHEF situated above β-BIDG (Figure 3c). Between βB/βC, βE/βF, βF/βG, and βH/βI, loops are inserted (BC loop, EF loop, FG loop, and HI loop) that form the exterior surface of the SpV4 capsid. In particular, the EF and HI loops are extensive surface loops with multiple subloops. These also contain four α-helices, αA, αB, αC in the EF loop, and αD in the HI loop, of which only αB and αD are surface accessible. The EF-loop subloops are primarily situated around the 2-fold symmetry axis of the capsid surface (Figure 4), with the exception of the subloop EF_4_ that forms the mushroom-like protrusion at the 3-fold symmetry axis (Figure 2). The HI subloops occupy the space between the icosahedral symmetry axes, whereas the FG loop forms the channel around the 5-fold symmetry axes (Figure 4). Lastly, the short BC loop is wedged between the EF and HI loops in the depressed region surrounding the 5-fold axis but occupies only a very small surface area.

### 3.4. The Dynamic Mushroom-like Protrusions

**Figure 5:**
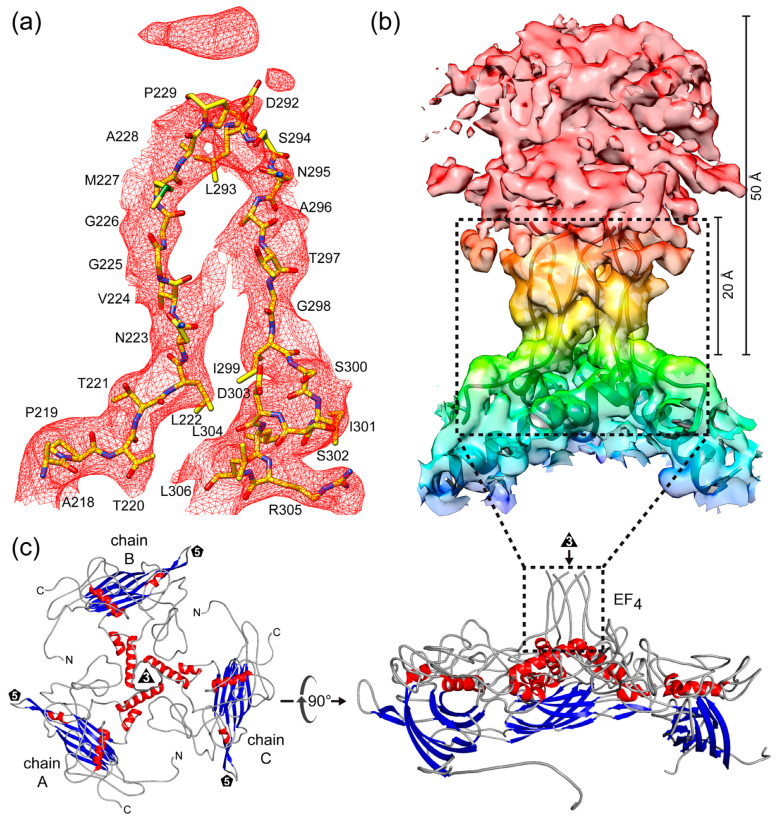
The mushroom-like protrusions of SpV4 VP1. (**a**) Modeled residues for surface loop EF_4_ (aa218–306) inside the SpV4 density map at a σ-threshold of 2. (**b**) Trimer of the EF_4_ loop arranged around the 3-fold symmetry axis inside the SpV4 density map. (**c**) Ribbon diagram of a SpV4 VP1 trimer centered down the 3-fold symmetry axis (**left**) and after a 90° rotation viewed from the side (**right**).

The most prominent feature of the SpV4 capsid is the mushroom-like protrusions that extend ~50 Å from the surface of the capsid (Figure 2c). These are formed by the EF_4_ loop at the 3-fold symmetry axis. In the cryo-EM density map, until glycine 226, the amino acid chain of the outward-going loop including their side chains were interpreted reliably. Starting with aa227, the map became less ordered and only the main chain was placed with confidence up to aa229. Additional amino acids were not built ~20 Å above the base of this loop, as this region is highly disordered with lower local resolution > 5 Å (Figure 5b and Figure S2a). In total, 62 aa of the EF_4_ loop (aa230–291) were not modeled. Mirroring the radially loop protrusion, only the main chain was interpreted for aa292–296 of the downward-going loop (Figure 5a). For threonine 297 and beyond, densities for both the aa main and side chains were reliably placed again. The mushroom-like protrusions in SpV4 are formed by trimers of the EF_4_ loops (Figure 5b). The base of this protrusion is formed by the αB-helix (aa300–321) surrounding the 3-fold axis (Figure 5c). A similar conformation was previously observed in the capsid of Aleutian Mink Disease Virus, a ssDNA virus of the *Parvoviridae* and the related phiEC6098 assigned to the same subfamily as SpV4 [8,34].

Increasing disorder with lower local resolution in areas protruding radially further from the center of the capsid is a common observation in cryo-EM maps (Figure S2a) [35]. Similarly, to SpV4, the *E. coli* phage phiEC6098 possesses the mushroom-like protrusions at the 3-fold symmetry axis that extend 70 Å from the capsid surface [8]. Due to its longer protrusion, 88 aa were not built in the model of the loop for phiEC6098. Protrusions such as these can result in disordered densities in cryo-EM maps because of movements or dynamic tilts of the distal ends of the protrusions relative to the remainder of the particle. Additionally, alternative loop conformations lead to diffuse densities during averaging of the sample of interest, preventing an effective interpretation of the structure.

**Figure 6:**
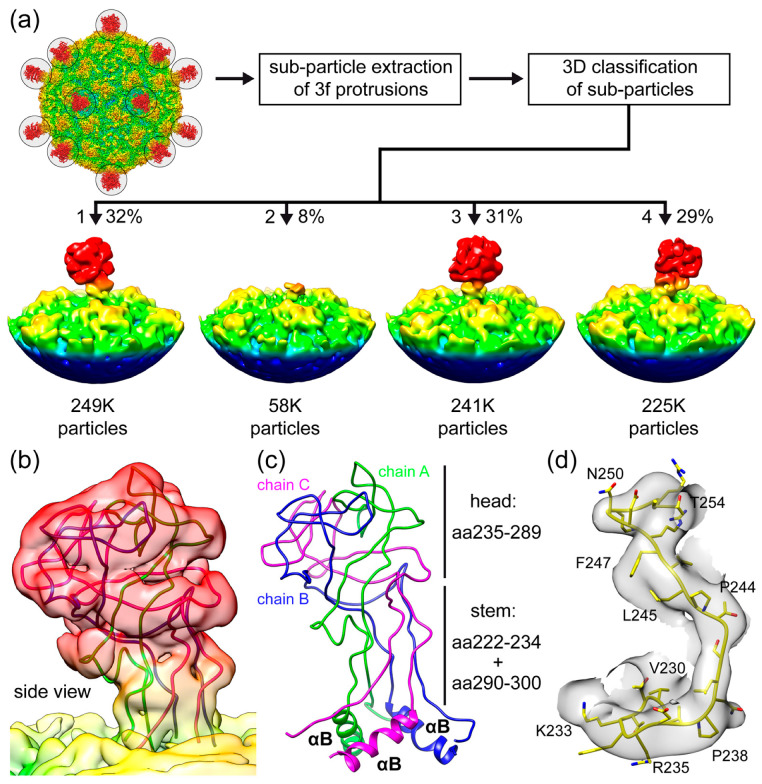
Heterogeneity of the mushroom-like protrusions. (**a**) Subparticles from the 3-fold axis are extracted and subjected to 3D classification. The individual 3D classes are shown with the number of particles assigned to each class listed below. (**b**) Fit of the EF_4_ protrusion model in the localized reconstructed map at a σ-threshold of 1.5 after the refinement of class 1 of the 3D classification shown from the side. (**c**) EF_4_ trimer, as shown in (**b**), where the aa ranges are provided for the head and stem regions. (**d**) Fit of the modeled aa230–254 in the localized reconstructed map at a σ-threshold of 2.

To obtain further structural information of SpV4’s mushroom-like protrusion, subparticles of the 3-fold region were extracted and subjected to 3D classification asking for four classes (Figure 6a). Following this procedure, the subparticles were assigned to the four classes. Class 1 (from ~32% of all particles), class 3 (~31%), and class 4 (~29%) showed the protrusion at the 3-fold symmetry axis of the SpV4 capsid. While the protrusions in the different classes lean towards different sides, they can be aligned by ±120° rotations. This tilt away from the 3-fold symmetry axis (Figure S2b) is the reason for the high disorder in the icosahedrally averaged cryo-EM map, as the loop does not conform to the icosahedral symmetry of the capsid. Interestingly, class 2 (~8% of particles) showed an absence of the protrusion altogether. When a model of a 3-fold-related SpV4 trimer was fitted into the density map of class 2, only the base of the loop up to aa222–224 and starting from aa295 to 298 were situated in the map, respectively. In the icosahedrally reconstructed map, a connected density between the upwards and downwards chain was observed in this region, indicating an alternative loop conformation potentially by a proteolytic cleavage of the loop (Figure 5a). If cleavages occur at the above-mentioned residues, VP1 would be segmented into a ~26 kDa N-terminal, a ~7 kDa EF_4_ loop, and a ~29 kDa C-terminal fragment, which could contribute to the weak bands observed in the SDS-PAGE (Figure 2a). Alternatively, high flexibility or an unfolded state of the protrusions could also result in the absence of observable density.

For a more detailed structural characterization of the protrusion, class 1 was further refined. Using a total of 249,227 particles, a density map was reconstructed to a nominal resolution of 3.7 Å (Figure 6b). However, local resolution estimations indicate that portions of the protrusion are of resolution > 4 Å (Figure S2b). This is likely the result of high flexibility of the loop. A trimer model of the SpV4 EF_4_ loop was predicted using AlphaFold 3 that generally fitted well in the density map [36]. In the absence of amino acid side-chain densities, only minor adjustments of the main chain for the head of the EF_4_ loop were needed (Figure 6b,c). In contrast, the region around the stem (aa220–239 and aa287–300) required major adjustments to agree with the transition into the body of the icosahedral capsid (Figure 6d).

Currently, a function of the EF_4_ protrusion is not known and protein function prediction tools such as DeepFRI are unable to identify any functions for this loop [37]. A potential function could be the attachment to their host, especially given the high sequence variability of this loop to other viruses in the subfamily. Alternatively, the protrusion could be associated with capsid assembly, as ~10% of the 3-fold regions appear to be absent of the loop. Other microviruses, such as phiX174, shed their scaffolding D-proteins upon maturation of the viral capsid [38]. In the absence of a D-protein homolog in SpV4, the EF_4_ protrusion could act in a similar fashion. More research is needed to understand the SpV4 receptor interaction and assembly process in similar detail as for phiX174. Hence, the elucidation of the SpV4 capsid structure in this study will aid future studies of the virus–host interaction, to understand disease mechanisms at a molecular level.

### 3.5. VP8 Is Observed on the Interior Surface of the SpV4 Capsid

**Figure 7:**
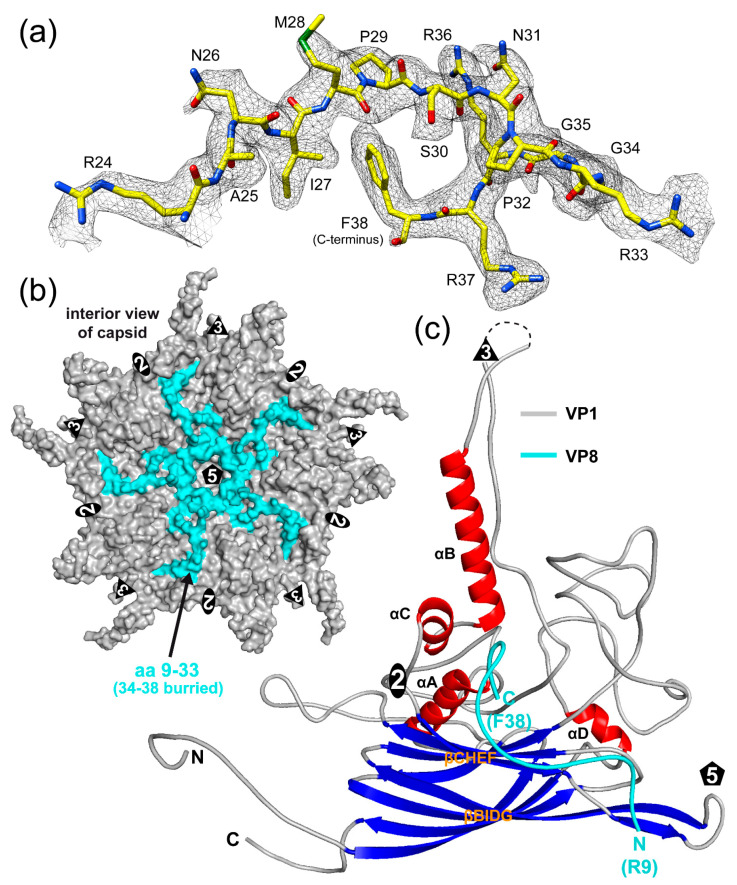
VP8 is observed on the interior side of the capsid. (**a**) Amino acid residues modeled for VP8 inside the SpV4 density map. (**b**) Pentamer surface representation viewed down the 5-fold axis from the interior side. VP1 is colored gray and VP8 is colored cyan. (**c**) Ribbon diagram of SpV4 VP1 and VP8. Please note that the model is rotated ~180° relative to Figure 3c.

**Figure 8:**
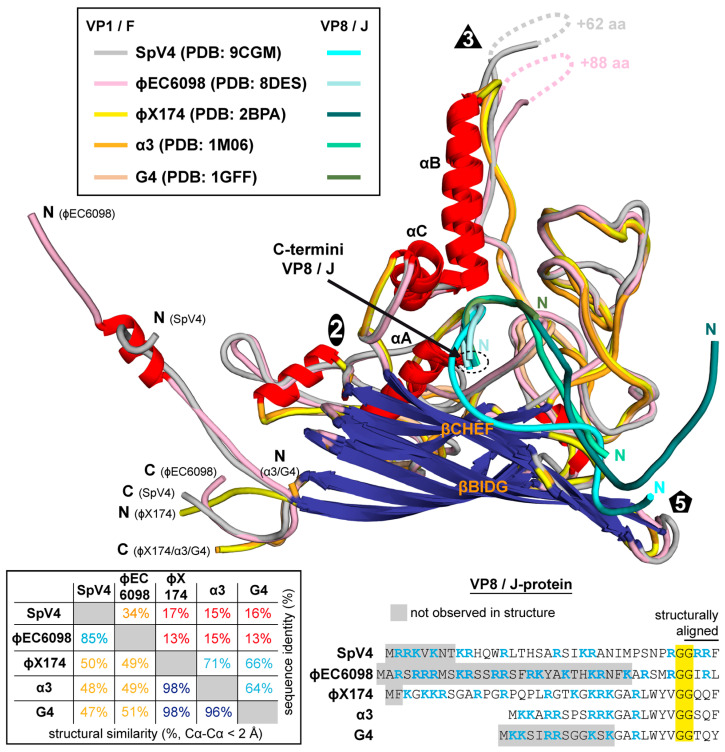
Structural comparison of the *Microviridae*. Superposition of the VP1/F-proteins of SpV4, phiEC6098, phiX174, bacteriophage α3, and G4 colored according to the legend. For the VP8/J-proteins, a different color scheme was utilized. The N- and C-termini are indicated. Bottom left: The amino acid sequence identity and structural identity for the viruses are provided. The structural similarity was defined as the percentage of aligned Cα atoms of the amino acid chain within 2 Å distance when the capsid structures were superposed. Bottom right: Amino acid sequence alignment of the VP8/J-proteins. Conserved aa are highlighted yellow and those in gray are not ordered in the structure. Basic residues are colored blue.

During the building of VP1, additional density on the interior side of the capsid was observed. For other structures of members of the *Microviridae*, a DNA-binding protein has been described to be located inside the capsid [7,8,19,20]. Due to the clear side-chain densities in this region of the map, the VP8 protein could be reliably built (Figure 7a). For this short, highly positively charged protein with a calculated pI of 12.9, structural order was observed for aa9–38. The N-terminal eight amino acids were not ordered but based on the first ordered residue, they should be located near the 5-fold axis (Figure 7b). Its C-terminal phenylalanine 38 is buried inside the capsid core, in a very highly hydrophobic region (Figure S3) near the 2-fold symmetry axis between β-CHEF, αA, and αB (Figure 7c). Additional contacts of VP8 to VP1 include several hydrogen bonds and salt bridges, primarily with its C-terminal region (aa33–38) and midsection (aa14–20) (Figure S3). The VP8 protein is also observed inside the empty capsids (Figure 2), with aa14–38 ordered. During phiX174 capsid maturation, the DNA-binding (J) protein guides the ssDNA for genome packaging inside the procapsid and remains in the interior of the capsid [38]. The fact that SpV4’s DNA-binding protein, VP8, is also observable in empty capsids could indicate that the empty capsids used to have a genome packaged but lost it during the capsid purification process or that the genome packaging process is different from phiX174. For the recently determined capsid structure of phiEC6098, only the C-terminal 10 aa of VP8 was ordered [8]. It was suggested that due to the smaller genome of phiEC6098 (~4.5 kb) relative to phiX174 (~5.4 kb), the N-terminal portion of VP8 which binds to the DNA is more flexible, as the packaged genome is less constrained inside the similar-sized capsid. However, SpV4’s genome is smaller (~4.4 kb) and the majority of VP8 is ordered, which contradicts this hypothesis. Alternatively, phiEC6098’s VP8 contains a total of 16 basic residues, whereas SpV4’s VP8 contains 11 basic residues in the non-buried portion of the protein, potentially making the former a stronger DNA binder (Figure 8).

### 3.6. Structural Comparison to Other Microviruses

The determination of the SpV4 capsid structure brings the total number of high-resolution capsid structures for the *Microviridae* to five. For their capsids (VP1 or F-protein), the aa identity ranges from 13 to 17% for members belonging to different subfamilies, but within the same subfamily, it is between 64 and 71% for the *Bullavirinae* (α3, G4, and phiX174) and 34% for the *Gokushovirinae* (SpV4 and phiEC6098) (Figure 8). Despite the low aa sequence identity, all these viruses exhibit the same overall capsid structure. They all share the core beta-barrel, the alpha helices A, B, and C, and the extensive EF and HI surface loops. Within the subfamily *Bullavirinae*, the capsid structures of α3, G4, and phiX174 are nearly identical. However, when the viruses of the different subfamilies and within the *Gokushovirinae* are compared, minor-to-major differences in these surface loops are observed. The most striking difference between the viruses of the *Bullavirinae* to the *Gokushovirinae* is the absence of the large insertion in the EF_4_ loop forming the mushroom-like protrusions. While most of this loop is not structurally well-ordered, this loop is also highly variable with regards to sequence identity and length for members within the *Gokushovirinae*, as shown here for SpV4 and phiEC6098. As mentioned above, the function of this loop is unknown but may be related to attachment to their host. While the members of the *Bullavirinae* do not have this extended loop, they possess a separate protein (G-protein) that interacts with the viral capsid and forms a pentamer around the 5-fold axis which helps the virion attach to the bacterial lipopolysaccharides [39]. Previously, a putative glucose binding site was identified in phiX174 near the α-helixB of the capsid (F-protein) [40]. In order to determine if SpV4 binds to any glycan structures, fluorescent-labeled SpV4 capsids were analyzed on a glycan array with ~600 different glycan molecules (Figure S4). While one glycan signal was above the background level, the overall binding level is low, indicating that either no binding glycan was present on the array or that SpV4 does not bind to glycans. The hosts of SpV4 are spiroplasmas, which are small cell-wall-deficient bacteria [41]. Currently, the presence of glycans has not been described for these bacteria. Another argument against the utilization of glycans for host recognition is that the receptors for chlamydia phages are proteinaceous in nature [42].

For the remaining surface loops, the highest divergence was observed in the EF_1_ and EF_3_ subloops that showed Cα-distances of >10 Å when SpV4 was compared to phiX174, α3, or G4. Additionally, Cα-distances of 5–10 Å were observed for the subloops EF_2_, HI_1_, HI_2_, HI_3_, and HI_4_. When SpV4 was compared to phiEC6098, Cα-distances of up to 5–8 Å were observed in EF_1_, EF_3_, and HI_5_ but, overall, these viruses are structurally much more similar.

Other regions of high structural variability for the *Microviridae* were the N- and C-termini and the DNA-binding protein (VP8/J-protein). At the VP1 N-termini, the *Gokushovirinae* display long extensions relative to the *Bullavirinae*. For phiEC6098, an α-helix has been described in this region interacting with VP8 [8]. In contrast, SpV4 does not form an α-helix in this region. Unlike phiEC6098, SpV4’s VP1 N-terminus makes a turn leading into the interior of the capsid. A straight conformation for the VP1 N-terminus, like for phiEC6098, is not possible as this region is occupied by VP8 that is more ordered in SpV4. Similarly, to VP1, SpV4’s VP8 N-termini leads under the 5-fold axis of the capsid. This is different from phiX174, where the N-terminus of the J-protein is located near the 2-fold axis. The C-termini of VP8 or the J-protein are located in the same, buried, mostly hydrophobic area of the capsid. One exception is phiEC6098, where one of the neighboring aromatic residues is changed to a glutamine (Q86) (equivalent to SpV4’s Y83, Figure S3). Only the four C-terminal DNA-binding protein residues are structurally conserved among all the analyzed viruses; they then diverge probably due to low sequence conservation (Figure 8). The VP8 of phiEC6098 is structurally more similar to SpV4’s VP8, with the seven C-terminal aa superposable. The N-terminal end of phiEC6098 VP8 leads further into the capsid interior, whereas SpV4’s VP8 lines the interior of the capsid with multiple interactions to VP1 (Figure S3), including to βF (extending the β-sheet) up to aa14. The N-terminal 14 aa also lead into the capsid interior but are not observed in empty capsids, which is further indicative of their role in DNA binding. Thus, despite structural differences, all these proteins contain several arginines and/or lysines residues and therefore will likely act as DNA-binding proteins.

### 3.7. Structural Repertoire of the Microviridae

**Figure 9:**
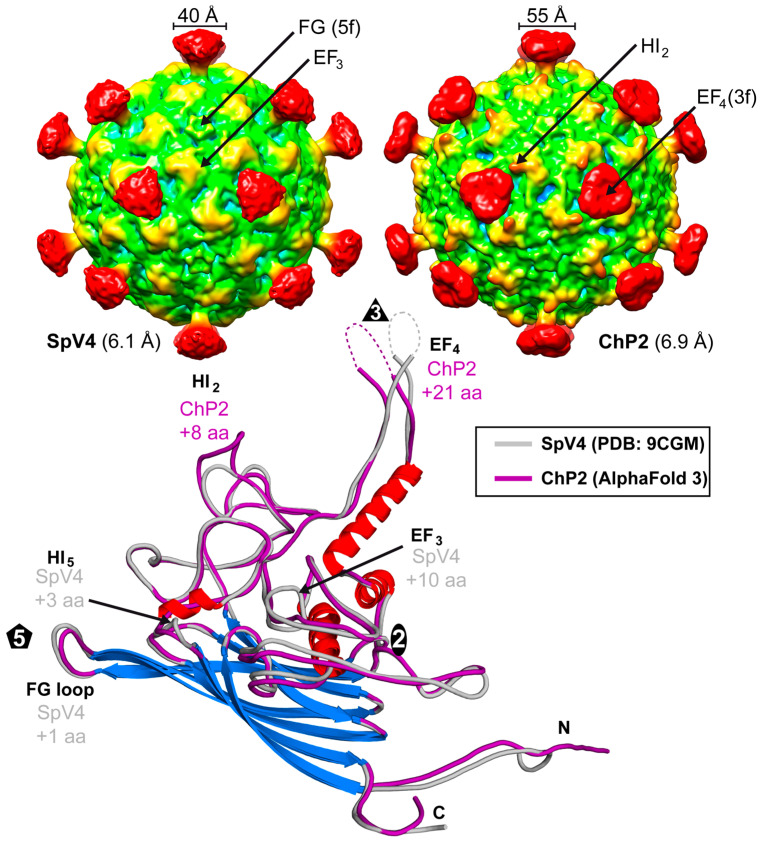
Low-resolution capsid structure of ChP2. The capsid surface density maps of SpV4 and ChP2 are contoured at a sigma (σ) threshold level of 1.0. Superposition of the SpV4 and ChP2 (predicted with AlphaFold 3) VP1 structure. Surface loops with insertions are indicated and their positions are shown in the cryo-EM maps above.

Structural comparison of the viruses of the *Microviridae* above indicated that the members of the *Gokushovirinae* are much more diverse than the *Bullavirinae*. Thus, another virus of this subfamily was analyzed. Chlamydia phage 2 (ChP2) specifically infects chlamydial bacterial strains that in turn infect humans and other hosts. The VP1 of ChP2 has a sequence identity of 34 and 54% to SpV4 and phiEC6098, respectively. Purified ChP2 samples were analyzed by cryo-EM and reconstructed to 6.9 Å resolution from 461 particles (Figure 9). A comparison of ChP2 capsids to SpV4 at a similar resolution showed broader mushroom-like protrusions for ChP2 (~55 Å). This is caused by a 21 aa insertion in the EF_4_ loop relative to SpV4. With this insertion, ChP2’s EF_4_ loop is only 5 aa shorter than phiEC6098, which has a more elongated folding (Figure 9). For further comparison, an AlphaFold 3 model of ChP2 was generated showing an additional 8 aa insertion in the HI_2_ loop, which can be seen in the cryo-EM as a more protruding area relative to SpV4 (Figure 9). In other loops, ChP2 consists of deletions compared to SpV4, including a 10 aa deletion in EF_3_, a 3 aa deletion in HI_5_, and a single aa deletion in the FG loop.

**Figure 10:**
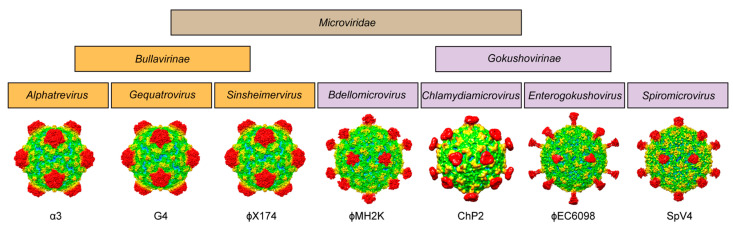
Family portrait of the *Microviridae*. All structures determined to date for the virus family are shown below their assigned genus. The one exception is phiMH2K, for which no structure has been determined yet and an AlphaFold 3 predicted model is shown.

For phiMH2K, a member of the genus *Bdellomicrovirus*, the VP1 shares ~30–50% sequence identity to the other members of the subfamily, with the highest homology to phiEC6098. However, phiMH2K has the shortest EF_4_ loop (4 aa shorter than SpV4) of all the viruses analyzed. All these analyses demonstrate the heterogeneity of the *Gokushovirinae*, which appears to be more diverse than the viruses in the *Bullavirinae* subfamily (Figure 10), suggesting that they may need to be further subdivided in the future.

## Figures and Tables

**Table 1:** Summary of data collection, image processing, and refinement statistics.

	SpV4 Full	SpV4 Empty
Micrographs	884	
Defocus range (µm)	0.5–3.0	
Electron dose (e^−^/Å^2^)	34	
Frames per micrograph	30	
Pixel size (Å/pixel)	1.06	
Particles used for final map	77,204	772
Resolution (Å)	2.52	3.02
Model refinement statistics		
Map CC	0.846	0.863
Residue range VP1	10–229, 292–553	20–229, 292–553
Residue range VP8	9–38	14–38
MolProbity Score	1.36	1.26
EMRinger Score	5.64	4.33
RMSD bond (Å)	0.01	0.01
RMSD angle (°)	0.91	1.18
All-atom clash score	5.15	4.67
Ramachandran (%)		
Favored	97.6	97.9
Allowed	2.4	2.1
Unfavored	0	0
Rotamer outliers (%)	0.4	0.4
C-β deviation (%)	0	0

## Data Availability

The SpV4 cryo-EM-reconstructed density map and model built for the capsid were deposited in the Electron Microscopy Data Bank (EMDB), with accession numbers EMD-45583 and PDB ID 9CGM.

## Supplementary Materials

The following supporting information can be downloaded at: https://www.mdpi.com/article/10.3390/v16071103/s1, Figure S1: Fit of SpV4 VP1 in the density map.; Figure S2: Local resolution in the cryo-EM maps.; Figure S3: SpV4 VP1 and VP8 interactions. Figure S4: Glycan array analysis of SpV4 capsids.
