# Supplementary material for: The Structure of Spiroplasma Virus 4: Exploring the Capsid Diversity of the Microviridae
Source: Viruses. 2024 Jul 9;16(7):1103. doi: 10.3390/v16071103 (PMC11281519; doi:10.3390/v16071103)
Supplement: Supplementary file 1 [file viruses-16-01103-s001.zip › viruses-3078299-supplementary.pdf]

Supplemental figures

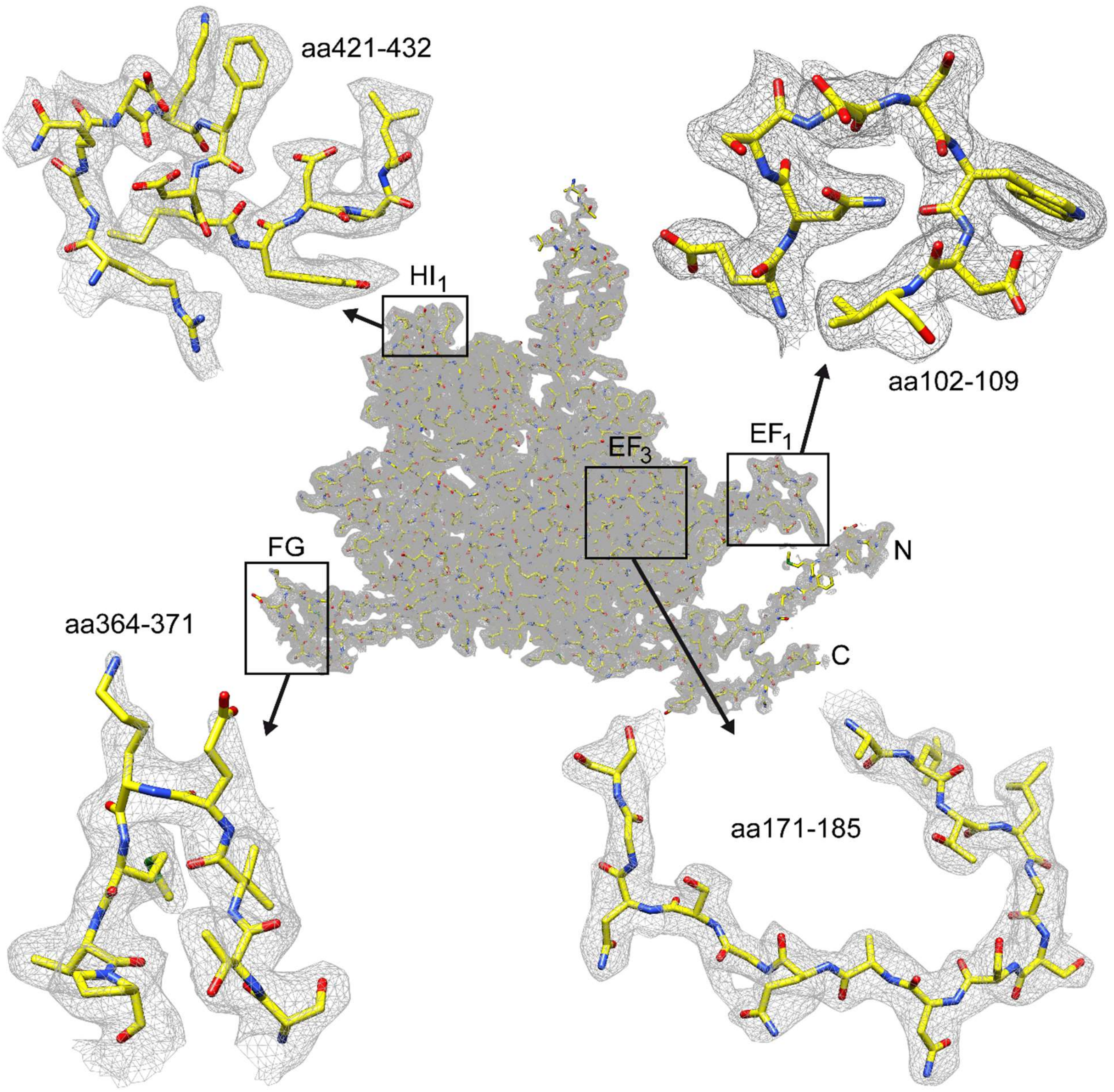

Figure S1. Fit of SpV4 VP1 in the density map. The full VP1 model was fitted into the density map (center). For several surface loops the local densities are provided showing the high resolution of the map. The amino acid residues are shown in stick representation and colored according to atom type: C = yellow, O = red, N = blue, S = green.

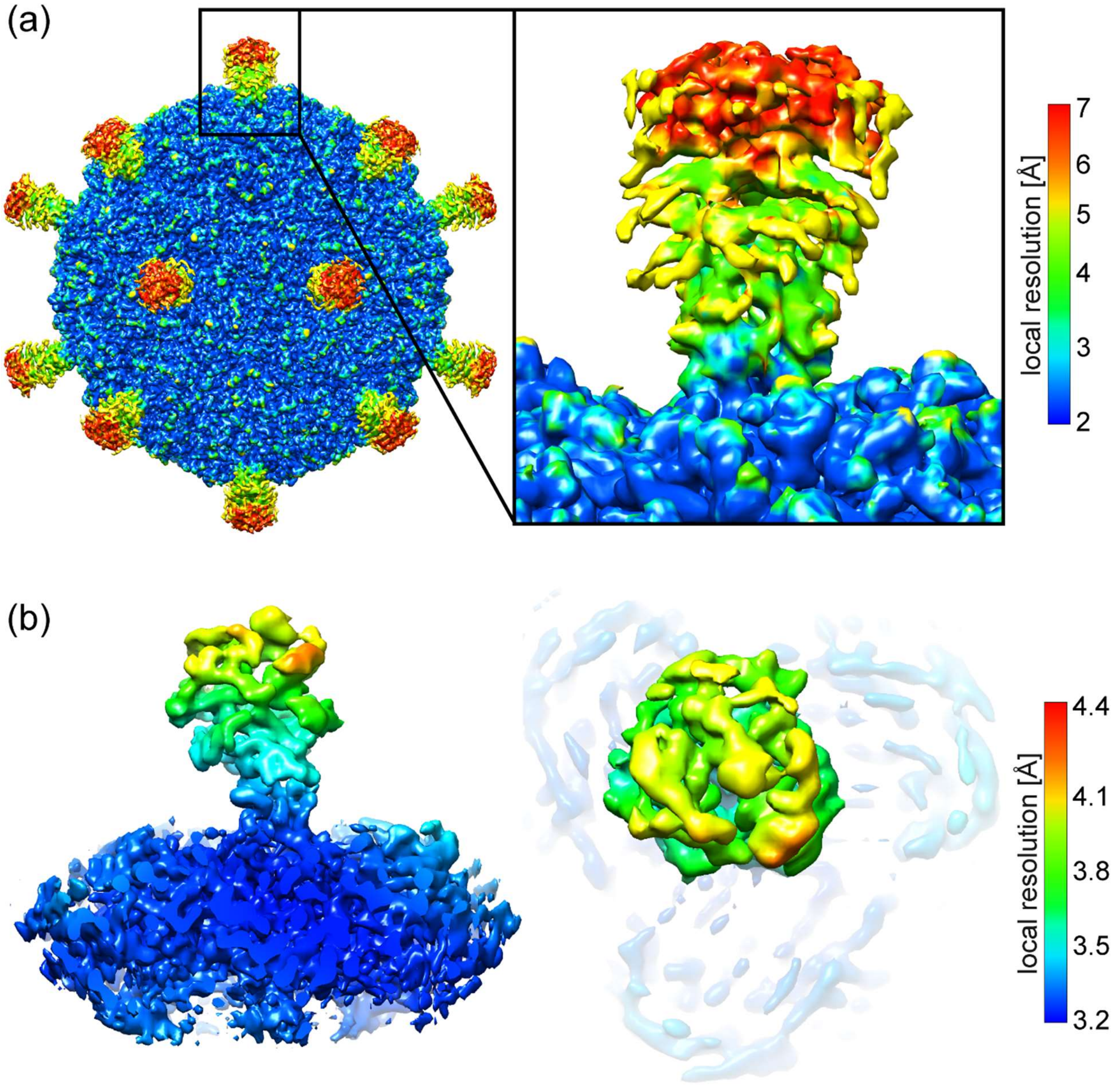

Figure S2. Local resolution in the cryo-EM maps. **(a)** The icosahedral-reconstructed map is displayed with the local resolution colored as determined by the ResMap application. A zoom in of the 3-fold protrusion shows that the local resolution is ~4-7 Å in this region, whereas the local resolution of the remaining capsid is between 2-3 Å resolution. **(b)** Analysis of the localized reconstruction as in (a) showing an improvement of the local resolution (3.5-4.2 Å) in the protrusion.

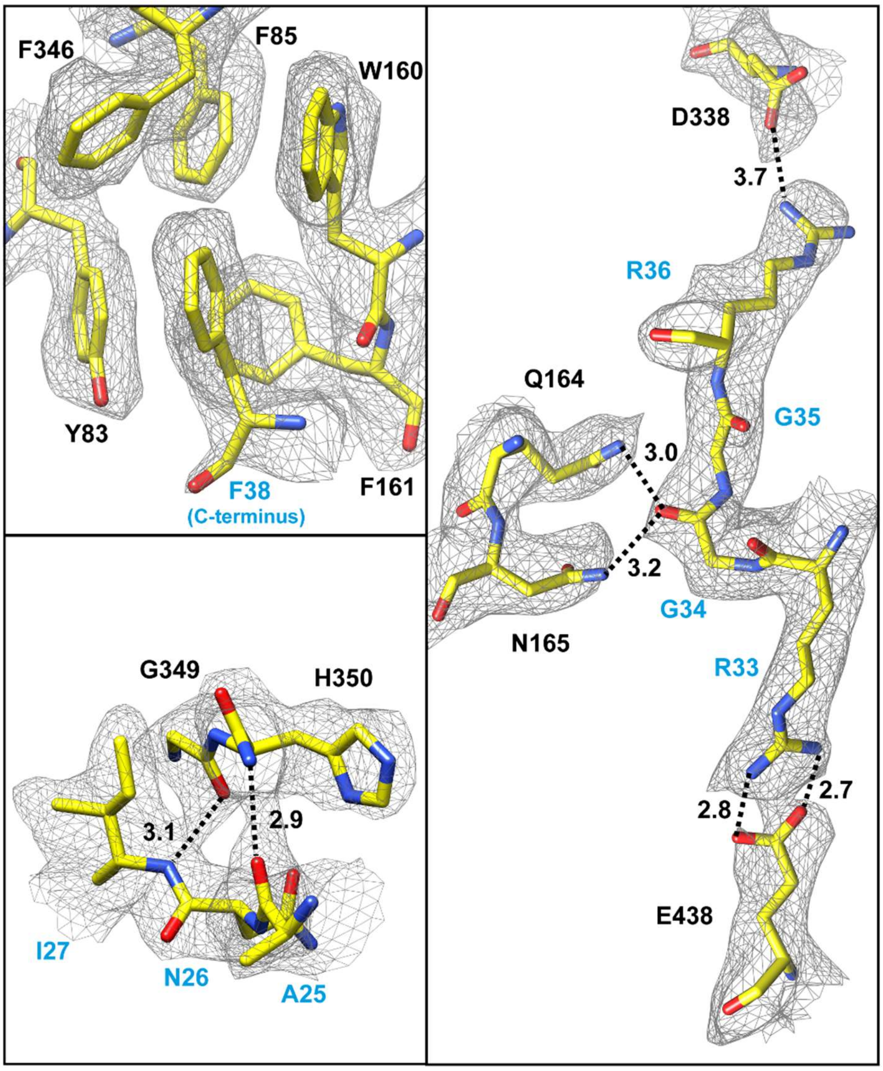

VP8 Residue (Atom)	VP1 Residue (Atom)	Distance (in Å)
R14 (O)	S358 (N)	2.9
T16 (O)	R66 (NH2)	2.6
H17 (NE2)	D77 (OD1)	3.1
H17 (ND1)	P74 (O)	3.2
S18 (OG)	M75 (O)	2.9
S18 (OG)	D531 (OD2)	2.6
A19 (N)	D531 (OD2)	2.8
R20 (NH2)	D76 (OD1)	3.3
A25 (N)	S351 (O)	3.0
A25 (O)	S351 (N)	2.9
I27 (N)	G349 (O)	3.1

VP8 Residue (Atom)	VP1 Residue (Atom)	Distance (in Å)
R33 (NH1)	E438 (OE2)	2.7
R33 (NH2)	E438 (OE1)	2.8
R33 (NE)	S209 (O)	2.9
G34 (O)	Q164 (NE2)	3.0
G34 (O)	N165 (ND2)	3.2
R36 (N)	E326 (OE2)	2.7
R36 (NH1)	D338 (OD2)	3.7
R36 (O)	R323 (NH1)	3.1
R37 (NH1)	T208 (O)	3.3
F38 (O)	R406 (NH2)	2.9

Figure S3. SpV4 VP1 and VP8 interactions. Shown are multiple examples of interactions including hydrophobic contacts, salt bridges, and hydrogen bonds. A comprehensive list of all hydrogen bonds is provided below.

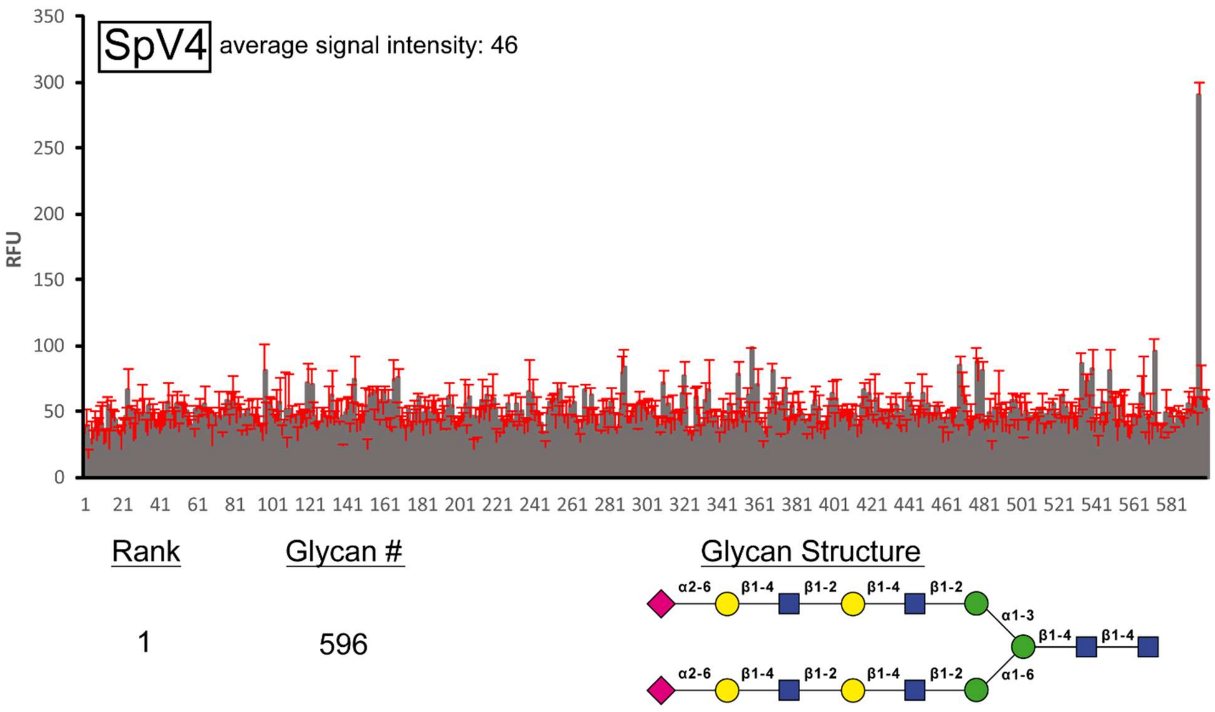

Figure S4. Glycan array analysis of SpV4 capsids. Relative fluorescence units (RFU) versus glycan number. 600 different glycan molecules (CFG glycan array v5.2) were screened with fluorescence-labeled SpV4 capsids. The capsids were labeled using the DyLight 488 antibody labeling kit (Thermo Fisher). The gray bars represent the fluorescence detected for each glycan with the standard deviation shown in red. Below a symbol representation of the glycan with the highest signals is provided. Blue square: N-Acetylglucosamine, green circle: mannose, yellow circle: galactose, pink diamond: N-Acetylneuraminic acid.
